# A Long-Term Efficacy Trial of a Live, Attenuated *Salmonella* Typhimurium Vaccine in Layer Hens

**DOI:** 10.3389/fmicb.2018.01380

**Published:** 2018-06-26

**Authors:** Andrea R. McWhorter, Kapil K. Chousalkar

**Affiliations:** School of Animal and Veterinary Sciences, University of Adelaide, Adelaide, SA, Australia

**Keywords:** *Salmonella* Typhimurium, *Salmonella* vaccine, layer hen, egg contamination, persistent infection, egg contamination

## Abstract

*Salmonella* remains one of the most common causes of bacterial foodborne gastrointestinal disease in humans. Raw eggs or food items containing undercooked eggs are frequently identified as the source of *Salmonella. Salmonella* Typhimurium contamination of table eggs most commonly occurs when they are laid in a contaminated environment. Several control strategies, including vaccination, are widely used to mitigate the total *Salmonella* load. It is unclear, however, whether live attenuated *Salmonella* vaccines are efficacious over the life span of a layer hen. Live attenuated *Salmonella* vaccines have been favored due to their ability to illicit a strong humoral immune response. The lifespan of a layer hen ranges between 60 and 80 weeks and the long term efficacy of attenuated vaccine strains has not been investigated. In this study, commercial brown layer chicks were vaccinated at day old, 6 weeks of age, and again at 10 weeks of age with the Bioproperties Vaxsafe^TM^ STM1 *aroA* mutant vaccine. Birds were challenged at 18 weeks of age with *Salmonella* Typhimurium DT9 (MLVA 03 15 08 11 550). Feces and eggs were monitored for *S.* Typhimurium for 40 weeks post-infection. Birds produced a strong immune response following the final dose which was administered intramuscularly. The serum antibody response to *S.* Typhimurium DT9 infection did not differ between challenged groups. Fecal shedding and egg contamination was highly variable and did not differ significantly between vaccinated and unvaccinated birds that had been challenged with *S.* Typhimurium DT9. Total bacterial load in feces was quantified using qPCR. No significant difference was detected between unvaccinated and vaccinated birds after challenge.

## Introduction

Foodborne salmonellosis is often epidemiologically linked with the consumption of *Salmonella* contaminated eggs or products containing raw or under cooked eggs (Threlfall et al., 2014). *Salmonella* contamination of eggs can occur through both vertical and horizontal mechanisms. Vertical transmission is primarily associated with *Salmonella* Enteritidis (*S.* Enteritidis) and occurs when the bacteria infects the oviduct of a hen subsequently contaminating egg internal contents during development (Gantois et al., 2009). Vertical transmission of other serovars including *Salmonella* Typhimurium (*S.* Typhimurium) has been documented but to a much lesser extent than *S.* Enteritidis (Keller et al., 1997; Okamura et al., 2001; Gast et al., 2011, 2013). Horizontal contamination occurs when an egg contacts a contaminated environment (Gantois et al., 2009). Bacteria can then adhere to the surface of the egg and migrate into eggshell pores (Gantois et al., 2009) or form biofilms on the shell surface (Pande et al., 2016b) representing a risk for downstream contamination of food items. In Australia, *S.* Enteritidis is not endemic on commercial poultry farms (OzFoodNet Working Group, 2015), thus, *S.* Typhimurium contamination of the layer hen farm environment represents an important public health issue.

The oral-fecal route of transmission is the primary mechanism through which layer hens become infected with *S.* Typhimurium on farm. Non-typhoidal *Salmonella* infection is often asymptomatic for adult birds and ultimately, the bacteria establish persistence and are continuously shed in feces (Pande et al., 2016a). Chronic shedding over the course of the productive life span of a layer hen can lead to a build-up of the bacteria in the shed environment. Bacterial titres as high as 10^5^ colony forming units (CFU) on eggshells collected from cage front have previously been observed (Gole et al., 2014).

Many on farm control measures such as strict farm biosecurity as well as routine chemical decontamination of farm equipment are widely employed to limit *Salmonella* contamination of eggs (De Cort et al., 2017). Methods to limit or prevent gastrointestinal colonization of hens are also commonly used and include the addition of organic acids to feed and water, use of probiotics and competitive exclusion products as well as vaccination (De Cort et al., 2017). Vaccination in combination with other control methods is viewed globally as an important strategy to reduce *Salmonella* in poultry, ultimately mitigating the foodborne risk of human disease (Desin et al., 2013). In the United Kingdom, a widespread vaccination program for layer hens has been associated with reducing human infection with *S.* Enteritidis (Cogan and Humphrey, 2003).

Several live attenuated vaccines are available worldwide for use in poultry. To date, the use of live attenuated vaccines has been favored because they elicit both cell mediated and humoral immune responses that provide protective immunity (Desin et al., 2013). Deletion of the metabolic gene, *aroA*, in *S.* Typhimurium, attenuated the virulence of this strain in mice and conferred protection upon challenge with a wild-type strain (Hoiseth and Stocker, 1981). Since, then several attenuated deletion mutant *S.* Typhimurium strains have been developed with the aim of reducing *Salmonella* shedding in poultry birds. Depending on the mutant bacterial strain, varying degrees of effectiveness have been observed (Barrow et al., 1990; Alderton et al., 1991; Hassan and Curtiss, 1997; Tan et al., 1997; Groves et al., 2011). These efficacy studies have largely focussed on the stimulation of a host immune response and changes in prevalence of the challenge strain. For a vaccine to be highly successful in a layer hen environment, it should also reduce the total bacterial load shed in feces, thus reducing the overall abundance of *Salmonella* in the environment in which eggs are laid. Furthermore, few studies in layer hens have trialed the effectiveness of live, attenuated vaccine strains over a commercially relevant period (Hassan and Curtiss, 1997; Groves et al., 2016).

In Australia, there is only one registered, commercially available *S.* Typhimurium vaccine. This vaccine is a live attenuated strain that was generated by disrupting the *aroA* gene through the insertion of the *Tn10* transposon (Alderton et al., 1991). Vaccination of poultry with the *aroA* mutant has been shown to elicit IgG, IgM, and IgA responses (Alderton et al., 1991) and has been shown to provide protection against wild-type challenge (Barrow et al., 1990; Alderton et al., 1991). Mode of inoculation has, however, been shown to have an effect on the strength of the antibody response and the protective effect of the vaccine (Groves et al., 2016). Currently, there is limited knowledge regarding the long-term efficacy of *Salmonella* vaccines at mitigating shedding over the productive life span of a layer hen, which can exceed 60 weeks of age. Furthermore, the correlation between changes in fecal shedding of *Salmonella* and bacterial egg contamination in vaccinated birds has not been clearly established.

In this study, commercial brown layer chicks were vaccinated at day old, 6 weeks, and again at 10 weeks of age with the Bioproperties Vaxsafe^TM^ STM1 *aroA* mutant vaccine. Birds were challenged at 18 weeks of age with *S.* Typhimurium. Feces and eggs were processed for isolation of *S.* Typhimurium for 42 weeks post-infection. PCR was also used for detection and quantification of *S.* Typhimurium in the feces. IgG and IgA antibody responses to vaccination and subsequent infection was also investigated.

## Materials and Methods

### Birds

Fertilized Hyline Brown eggs (*n* = 110) were obtained from a commercial breeding company. Parent flocks had not received the *S.* Typhimurium vaccine. Eggs were fumigated with formaldehyde to kill any bacteria present on the egg shell surface and incubated at 38°C over a period of 21 days. The relative humidity was maintained at 45–55% till day 18 and then increased to 55–65% until hatching. Chicks began hatching at day 21 producing 62 males and 36 females. Males were separated and included in a separate experiment. Meconium samples were collected and tested for *Salmonella* using previously described isolation methods (Gole et al., 2014). On day 1 post-hatch, female chicks (*n* = 36) were divided into vaccinated and non-vaccinated groups and housed in a positive pressure animal facility at the Roseworthy Campus, University of Adelaide. At 12 weeks of age, birds were separated into 4 groups [control (*n* = 5), vaccinated control (*n* = 5), unvaccinated challenge (*n* = 12) and vaccinated challenge (*n* = 14), and housed in separate rooms]. The sample size for treatment groups was determined using OpenEpi (Sullivan et al., 2009) with a 95% confidence interval and an 80% power level for detecting statistical significance between challenged only and vaccinated and challenged treatment groups, with α = 0.05. During rearing, samples were collected fortnightly and processed to detect STM1 vaccine and wild-type *Salmonella* by culture.

All rooms within this facility had been previously decontaminated with SaniGuard (Chemetall, Australia). All animal pens, cages, trays, feeders, laboratory equipment, as well as the floor and walls of the rooms had previously been cleaned extensively with FoamCleanS (Chemetall, Australia) and SaniGuard (Chemetall, Australia). All staff wore sterile, disposable overalls, head-covers, shoe covers, masks and gloves while working within the facility. Feed was disinfected by fumigation with formaldehyde. Water was disinfected by the addition of chloride tablets. Once the birds reached 60 weeks of age, the trial was terminated. All housing procedures and experiments were performed according to animal ethics protocol approved by the University of Adelaide (UA) Animal Care and Use Committee, S-2015-228.

### Vaccination Schedule

The vaccination schedule used in this study was recommended by Bioproperties and is the current practice used by Australian egg producers (Bioproperties, personal communication). At day 1 post-hatch, birds in the vaccination treatment group received the first vaccination. The Vaxsafe ST (*S.* Typhimurium strain STM1, Batch Number STM142921B) vaccine (Bioproperties, Australia) was diluted in 0.9% saline. A total dose of 1 × 10^7^ CFU was administered to each chick by dropping 25 μl of diluted vaccine into each eye using a sterile pipette. Birds in the control, non-vaccinated group received 25 μl of 0.9% saline into each eye.

The second dose of the STM1 vaccine was administered at 6 weeks of age. Vaccine was diluted to 5 × 10^6^ CFU/ml in 2 L of autoclaved tap water and provided to birds *ad libitum* over a 24 h period. Birds consumed a total of 700 ml of water containing the STM1 vaccine, giving an average dosage of 10^9^ CFU/bird. Control, non-vaccinated birds received only autoclaved tap water during the same period.

At 10 weeks of age, birds were given the final dose of vaccine. STM1 was reconstituted in Marek’s diluent. Birds were inoculated with 200 μl of STM1 (1 × 10^7^ CFU) vaccine into the right pectoralis muscle.

### Bacterial Challenge

A *S.* Typhimurium definitive type (DT) 9 (MLVA 03 15 08 11 550) isolate was selected for use in this study. This isolate had been previously isolated from layer farm and was serotyped by the *Salmonella* Reference Laboratory, Institute of Veterinary Medical Science (IMVS), Adelaide, SA, Australia. Long-term frozen stocks of this isolate were maintained at -80°C. Prior to use, bacteria were resuscitated by streaking on to nutrient agar.

To generate the challenge inoculum, a single *S.* Typhimurium DT9 colony was added to 5 ml of Luria Bertani (LB) broth and incubated at 37°C with shaking (110 rpm) for 6 h. Subsequently, 10 μl of this culture was added to 10 ml of fresh LB broth and incubated under the same conditions overnight. The amount of bacteria in the broth was determined by measuring the optical density at 600 nm using a spectrophotometer. Bacteria were diluted in LB to achieve the desired concentration. A challenge dose of 1 × 10^9^ CFU *S.* Typhimurium DT9 was selected for this study based on results previous studies (Tan et al., 1997; Pande et al., 2016a).

At point-of-lay (18 weeks of age), 14 hens from the vaccinated and 12 from the challenge only groups were administered with 1 × 10^9^ CFU *S.* Typhimurium DT9 in a 1.0 ml inoculum by oral gavage. Birds in the control and vaccinated only treatment groups received 1.0 ml of sterile LB broth. For the first 48 h post infection (p.i.), birds were monitored twice daily.

### Fecal and Egg Sampling

Feces and eggs were collected from individual birds on days 1, 3, 6, 9 12, and 15 p.i. and then weekly until week 21 p.i. when sample collection changed to fortnightly until the end of the experiment. The day prior to sampling, clean plastic sheets were placed underneath individual cages to facilitate the collection of fresh fecal material from each bird. Samples were returned to the lab and processed immediately for the presence of *S.* Typhimurium DT9 and/or STM1 using a previously described *Salmonella* enrichment culture method (Gole et al., 2014). *S.* Typhimurium DT9 and STM1 vaccine are easily distinguishable from each other on xylose lysine deoxycholate (XLD) agar plates. The STM1 vaccine strain does not produce hydrogen sulfide and colonies appear pink when cultured on XLD.

To determine the level of contamination on the egg shell surface, up to three eggs from each bird laid during the week, were placed into a single, sterile, resealable bag and 10 ml/egg buffered peptone water was added to each bag. Eggs were massaged for 90 s and the buffered peptone water was subsequently placed into a sterile 25 ml container. Eggs were then placed into a container filled with 70% ethanol for 90 s and air dried. Internal contents were collected by cracking the egg into a sterile bag. Egg contents were mixed thoroughly and then 3 ml was placed into a container filled with 20 ml of buffered peptone water. *Salmonella* culture continued as above.

### Detection of Salmonella IgG and Mucosal IgA Antibodies by ELISA

Blood samples were collected (∼1 ml) from chicks 2 weeks after each dose of the STM1 vaccine. Following challenge with *S.* Typhimurium DT9, blood was collected (∼3 ml) from each bird, weekly and then fortnightly from week 21 p.i. All blood samples were collected into lithium-heparinized vacutainers (Becton Dickenson, United States). Samples were centrifuged at 2348 *g* for 10 min. Plasma was collected with a pipette and transferred into a clean, microcentrifuge tube. Samples were frozen at -20°C.

*Salmonella* group B antibody was quantified using the BioChek LPS Group B chicken *Salmonella* antibody test kit (Veterinary Diagnostics). The ELISA and antibody titre calculations were conducted as per manufacturer’s instruction. Samples were diluted 1:100 with two replicates.

The antibody avidity assay used was described previously by Ross et al. (2000). In this experiment, plasma collected 10 days after the final administration of the STM1 vaccine, week 3 p.i., and week 39 p.i. were processed in triplicate and diluted 1:100 before addition to the BioChek LPS ELISA plate. The relative avidity was tested by adding 0, 0.5, 1.5, 2.0, 2.5, or 3.0 molar concentrations of the chaotrophic agent, sodium thiocyanate.

Secretory IgA was measured using chicken IgA ELISA kit (Abcam ab157691). Mucosal scrapings from the distal ileum were collected and frozen at -80°C until ready for use. Mucus samples were thawed, weighed, and suspended in 4 volumes (wt/wt) of phosphate buffered saline (PBS). Samples were mixed thoroughly and then centrifuged at 5,000 *g* for 10 min. The IgA ELISA was conducted as per the manufacturer’s instruction with minor modification. Mucosal samples were diluted 1:2000. The ELISA was read at 450 nm using a ClarioStar plate reader (BMG LabTech).

### Tissue Processing for Bacteriology

At 60 weeks of age, birds were humanely euthanized by Lethabarb (Virbac, Australia) injection following the manufacturer’s dosing recommendation by a licensed veterinarian. Organs were collected aseptically for culture and molecular detection of *S.* Typhimurium DT9 and the STM1 vaccine strain. Biopsy punches (5 mm^2^) were collected for ileum, caecum, infundibulum, isthmus and vagina. Because of the thick basement membrane, sections of the magnum and shell gland were cut with a sterile scissors. Small sections of liver and spleen were also collected. Cut samples were weighed. Tissues were homogenized with sterilized, stainless steel beads 0.5 – 2.0 mm in 0.5 ml of 0.9% saline. Tissues were homogenized using a Bullet Blender (Next Advance, United States) on full speed for 5 min. If necessary, an additional homogenization was performed to ensure the tissue was completely disrupted. 100 μl of the tissue homogenate was then diluted in to 900 μl of sterile 0.9% saline and diluted again 1/10. Ten microliters of each dilution was drop plated onto XLD agar (ThermoFisher Scientific, Australia) and incubated overnight at 37°C. Colonies of both wild-type *S.* Typhimurium and STM1 were enumerated. Strains were distinguished as indicated above.

To enrich the sample, 100 μl of each tissue homogenate was also placed in to 10 ml of buffered peptone water. Samples were incubated overnight at 37°C and then streaked out on to XLD agar. Agar plates were incubated at 37°C overnight and then plates were scored for the presence of *S.* Typhimurium DT9 or the STM1 vaccine.

### Standard PCR Detection of *S.* Typhimurium DT9 and STM1 Vaccine

Total DNA was purified from fecal and cecal samples using the Isolate Fecal DNA kit (Bioline, Australia) with some modifications. Briefly, 150 mg of sample was added to a safelock microcentrifuge tube (Eppendorf, Australia) containing beads from the kit. Lysis buffer was then added and samples were homogenized using a Bullet Blender tissue homogenizer at full speed for 5 min. Tubes were then incubated at 80°C for 30 min and then cooled to room temperature. Samples were then vortexed to mix. The remaining DNA purification procedure was conducted as per manufacturer recommendation.

The Promega Wizard Genomic DNA kit was used to purify DNA from tissues with some modification. Briefly, 200 μl of tissue homogenate was combined with 600 μl nuclei lysis solution and 17.5 μl of 20 mg/ml Proteinase K (Promega, Australia). Samples were incubated overnight at 55°C. The remainder of the DNA purification procedure was performed as per manufacturer instruction.

To detect the vaccine strain, primers (Forward 5′-3′GTTTTAAGTGTAATTCGGGG; Reverse 5′-3′ TATGATCAAATGGTTTCGCC) were designed to the transposon/*aroA* gene junction unique to the Vaxsafe STM1 vaccine strain. This PCR reaction generated an amplicon of 164 base pairs. To differentiate wild-type *Salmonella* Typhimurium, primers were designed to the region including the *aroA* gene junction which is conserved for *Salmonella* strains (Forward: 5′-TCTTTTTTCATCCCCACG-3′; Reverse: 5′-CGGTTTTACCACAAGCTAA-3′). The *aroA* gene junction sequence from four S. Typhimurium isolates was aligned to ensure primer specificity. A BLAST search was then conducted to identify potential cross reactivity of the primers with a non-specific target. The top 100 hits for both primers were *Salmonella* sequences. Lastly, to confirm primer specificity, the wild-type *Salmonella* PCR was tested against 20 different wild type *Salmonella* serovars, the STM1 vaccine, and one strain of *Escherichia coli* (data not shown). All wild type *Salmonella* serovars generated a 182 bp amplicon (data not shown). No amplicon was observed for the STM1 vaccine or the *Escherichia coli* (data not shown).

Both wild-type and vaccine specific PCR reactions were conducted using a total volume of 20 μl. Each reaction contained 20 ng DNA, 1x My Red Taq reaction buffer, 2 mM each forward and reverse primer, 0.2 units of My Red Taq polymerase (Bioline, Australia) and nuclease free water. PCR cycling conditions were the same for both reactions and were performed using a Biorad T100 thermocycler. The first step was an initial melt at 95°C for 3 min. The second step included 40 cycles of 95°C for 30 s, 60°C for 30 s, and 72°C for 1:00. A final extension was done at 72°C for 5 min. Both standard PCR reactions had a limit of detection of 10^2^ CFU.

### Quantitative PCR Assessment of Fecal Samples for Wild-Type *Salmonella*

Using the wild-type *Salmonella* specific primers listed above, a qPCR was designed to quantify the bacterial load in fecal samples that had tested positive for *S.* Typhimurium DT9 using standard PCR. The total reaction volume was 10 μL and contained 10 ng sample DNA, 5 μL 2x Quantifast SYBR Green Master Mix, and 1 μM of both forward and reverse primers. The qPCR was performed with the Quantifast^®^ SYBR^®^ Green qPCR kit (Qiagen, Australia) as per manufacturer instruction. Serial ten-fold dilutions of *S.* Typhimurium DT9 spiked fecal samples were used to generate a standard curve and determine the limit of detection (≥1000 of *Salmonella* CFU/g of feces). Negative and positive controls were included in every reaction. A two-step qPCR cycling method was used as per (Sharma et al., 2017). Cycling conditions included an initial melt at 95°C for 5 min followed by 35 cycles of 95°C for 10 s and 58°C 30 s using a Rotor Gene 6000 (Corbett Research, United States). Negative samples were assigned a limit of detection value of 1000 CFU/g feces.

### Statistics

Two-way analysis of variance (ANOVA) and subsequent Tukey’s comparisons tests were used to determine if vaccination had an effect on prevalence (bacteriological culture) or abundance (qPCR) of *S.* Typhimurium DT9 in feces over the experimental time period. The same analysis was used to characterize the effect of vaccination on the prevalence of *S.* Typhimurium DT9 contamination of eggs collected throughout the experiment.

A D’Agostino and Pearson test of normality was conducted on all antibody titre data. All data sets were normally distributed. Un-paired *t*-tests were performed to determine if there were significant differences in IgG titres obtained from serum collected from vaccinated and control chicks after each dose. Two-way ANOVA and Tukey’s comparison of the means was used to analyze antibody titres over the experimental time course from all treatment groups. A one-way ANOVA and Tukey’s comparison test was used to determine if vaccination had an effect on the concentration of secreted IgA in ileal mucosal scrapings. *P*-values less than 0.05 were considered statistically significant.

## Results

### Pre-challenge Phase

At hatch, meconium samples collected from chicks were culture and PCR negative for *Salmonella*. One week after the first vaccine dose, 10 of 12 (83.3%) fecal samples collected from the rearing pen of vaccinated birds were positive for STM1. One week following the second oral dose of STM1 vaccine in drinking water, 8 of 8 (100%) samples collected from the vaccinated group were positive for STM1 vaccine. No clinical symptoms were observed following each administration of the STM1 vaccine.

Fecal samples collected from all birds during the rearing, pre-challenge phase of this study remained *Salmonella* negative.

### Evidence of Disease Post-challenge

Up to 6 h post-infection (p.i.) with *S.* Typhimurium DT9, birds did not exhibit any clinical symptoms of disease. After 24 h, mucoid and blood tinged feces were observed for the challenged only group at a higher frequency than birds that had been vaccinated and challenged (**Table 1**).

**Table 1:** Frequency of mucoid or blood tinged feces in *S.* Typhimurium DT9 challenged birds.

Days post infection	Challenged Only				Vaccinated and Challenged			
	Appearance of feces				Appearance of feces			
	Mucoid		Blood		Mucoid		Blood	
	Frequency	Percent	Frequency	Percent	Frequency	Percent	Frequency	Percent
1	5/12	41.7	2/12	16.7	2/14	14.3	0/14	0
2	7/12	58.3	2/12	16.7	9/14	64.3	0/14	0
3	12/12	100	8/12	66.7	4/14	28.6	0/14	0
4	11/12	91.7	8/12	66.7	3/14	21.4	0/14	0
5	6/12	50	1/12	8.3	0/14	0	0/14	0
6	4/12	33.3	0/12	0	0/14	0	0/14	0
7	0/12	0	0/12	0	0/14	0	0/14	0

All birds in the challenge only group exhibited hunching behavior for 3 days p.i. From day 4 p.i., normal feed and water consumption were observed and birds exhibited normal physical activity. No hunching or reduced feed/water consumption was observed in the vaccinated and challenged group. When challenged, the birds were at point-of-lay, thus any effects on egg production were difficult to identify.

Control (*n* = 5) and vaccinated only (*n* = 5) birds were not challenged and did not exhibit any clinical signs of disease. These birds remained negative for *S.* Typhimurium DT9 over the course of the entire 60 week experiment. It should also be noted that the STM1 vaccine strain was not cultured from either of these treatment groups.

### Shedding of *S.* Typhimurium DT9 and STM1 Vaccine in Feces

**FIGURE 1:**
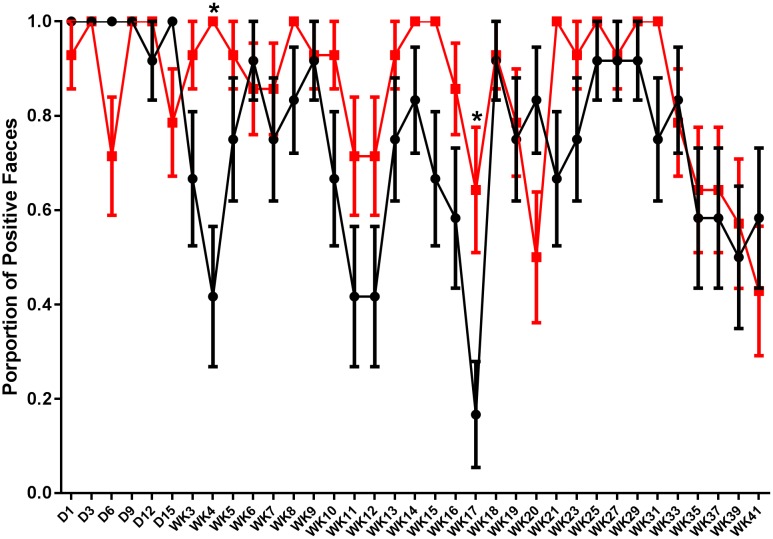
Shedding pattern of *S.* Typhimurium DT9 in feces. Fecal samples were collected and tested for the presence of *S.* Typhimurium DT9 using standard culture methods over a 41 week period. Data are presented as mean proportion of *S.* Typhimurium positive fecal samples ± the standard error. Samples collected from challenged only (black line) as well as vaccinated and challenged (red line) birds were positive for *S.* Typhimurium. A two-way ANOVA test revealed significant effects of both treatment and time (*P* < 0.0001). A significant interaction was also observed between both factors (*P* = 0.018). Multiple comparison of the means revealed the proportion of positive feces in vaccinated and challenged birds was significantly greater proportion than challenged only birds at weeks 4 and 17 p.i.

Fecal shedding of *S.* Typhimurium DT9 was monitored using *Salmonella* culture methods on days 1, 3, 6, 9, 12, and 15 p.i. and then weekly until week 21 p.i. when sampling was performed fortnightly until the end of the experiment. Data are presented as the proportion of individuals with *S.* Typhimurium DT9 positive feces ± the standard error of the mean. The number of fecal samples positive for *S.* Typhimurium DT9 was variable for both the challenged only and vaccinated and challenged treatment groups (**Figure 1**). Significant effects of treatment (challenged only verses vaccinated and challenged) and time were observed (*P* < 0.0001; df = 34) and an interaction was observed between both factors (time and treatment) (*P* = 0.018). A *post hoc* comparison of the mean proportion of positive feces revealed that vaccinated and challenged birds exhibited significantly greater proportion of birds shedding *S.* Typhimurium DT9 at both weeks 4 (*P* < 0.01) and 17 p.i. (*P* < 0.05) (**Figure 1**).

**FIGURE 2:**
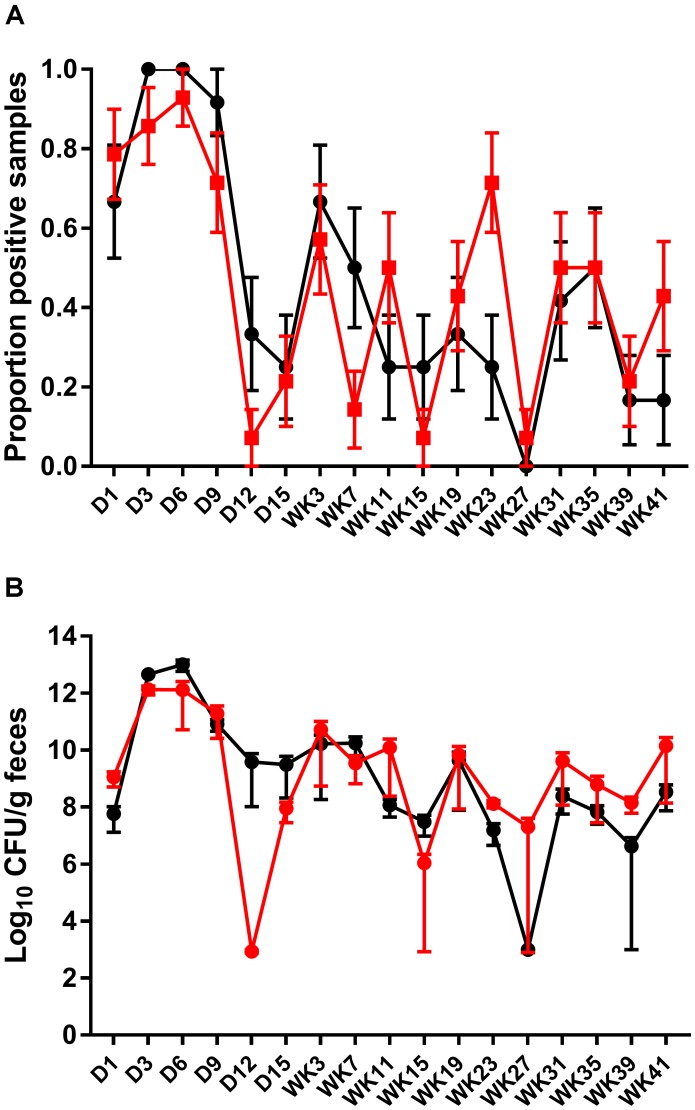
Detection and quantification of *S.* Typhimurium DT9 by PCR. The prevalence of *S.* Typhimurium shedding was characterized using standard PCR **(A)**. Consistent with culture results, *S.* Typhimurium positive samples were highly variable over the experimental time course. No significant difference in the proportion of positive fecal samples was observed. The abundance of S. Typhimurium present in fecal samples collected from both challenged only and vaccinated and challenged birds was determined by qPCR **(B)**. Peak *S.* Typhimurium DT9 load in feces was observed at days 3 and 6 p.i. No significant difference in bacterial load was observed between the two treatment groups.

Fecal shedding of *S.* Typhimurium DT9 was also monitored by standard PCR. The limit of detection of the PCR reaction was 10^2^ CFU (data not shown). As with culture methods, the proportion of fecal samples positive for *S.* Typhimurium DT9 was highly variable over the experimental time course (**Figure 2A**). A significant effect of time was detected (*P* < 0.001, df = 16) but there were no significant differences between treatment groups (vaccinated verses unvaccinated). At no time point was there a significant difference in the proportion of positive fecal samples as detected by PCR.

A wild-type *Salmonella* qPCR reaction was also conducted to determine whether vaccination had an effect on the total abundance of *S.* Typhimurium DT9 shed in fecal samples. Peak *S.* Typhimurium DT9 load in feces was observed at days 3 and 6 p.i. in both challenged only and vaccinated and challenged groups (**Figure 2B**). Bacterial loads in both challenged only and vaccinated and challenged treatment groups were significantly reduced over time (*P* < 0.01, df = 16). No significant effect of vaccination, however, was observed on the total abundance of *S.* Typhimurium DT9 in fecal samples (**Figure 2B**).

Fecal samples collected from the vaccinated and challenged treatment group were also monitored for the presence of the STM1 vaccine strain using *Salmonella* culture methods (**Table 2**). Shedding of STM1 vaccine was variable from day1 p.i. to week 3 p.i. Peak shedding was observed at day 3 p.i. when 100% of the birds in the vaccinated and challenged group tested positive for STM1. After week 3 p.i., STM1 vaccine was not detected in feces.

**Table 2:** Proportion of fecal samples positive for the STM1 vaccine strain post challenge.

	Day 1	Day 3	Day 6	Day 9	Day 12	Day 15	Week 3
STM1 positive feces	8/14 (57.1%)	14/14 (100%)	1/14 (7.1%)	4/14 (28.6%)	2/14 (14.3%)	0/14 (0%)	4/14 (28.6%)

Detection of STM1 vaccine in feces was also conducted using STM1 standard PCR. Detection of STM1 vaccine was highly intermittent. On day 9 and week 7 p.i., one out of 14 fecal samples tested positive for STM1. STM1 positive samples were also observed at weeks 19 (2/14), 23 (4/14), and 39 (2/14) p.i.

### Monitoring Egg Shells and Internal Contents for *S.* Typhimurium DT9 and STM1 Vaccine

**FIGURE 3:**
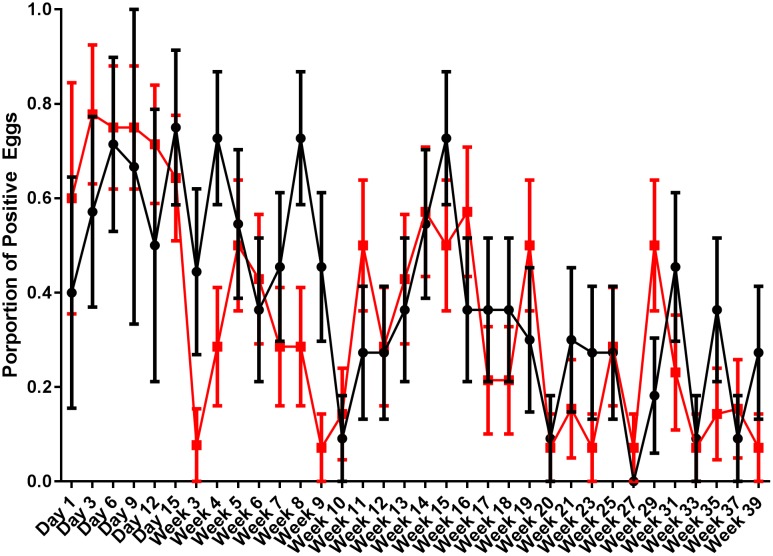
Proportion of egg shells positive for *S.* Typhimurium DT9. Egg shells were washed with buffered peptone water which was then cultured for *S.* Typhimurium DT9. Data are presented as the proportion of egg shells positive for *S.* Typhimurium DT9. The number of positive egg shells was variable for both treatment groups. A significant effect of time was detected (*P* < 0.001). No significant difference was observed between treatment groups.

Egg shells were processed for the presence of *S.* Typhimurium DT9 and STM1 vaccine over the same time course as fecal samples. Data are presented as the proportion of eggs testing positive for *S.* Typhimurium DT9 by culture (**Figure 3**). As with fecal samples, the proportion of egg shells positive for *S.* Typhimurium DT9 was highly variable over the course of the entire experiment. A significant effect of time was detected (*P* < 0.001, df = 33) but no significant effect of treatment was observed (*P* > 0.05, df = 33). The total number of *S.* Typhimurium DT9 positive egg shells was, in general, lower than fecal samples.

STM1 vaccine was not detected at any time point during the experiment on egg shells from either treatment group.

Egg internal contents were aseptically collected and tested for both STM1 vaccine and *S.* Typhimurium DT9. Over the course of the experiment 3,297 eggs were processed and all egg internal contents were negative for both *S.* Typhimurium DT9 and STM1 vaccine.

### IgG Response Pre- and Post-challenge With *S.* Typhimurium DT9

**FIGURE 4:**
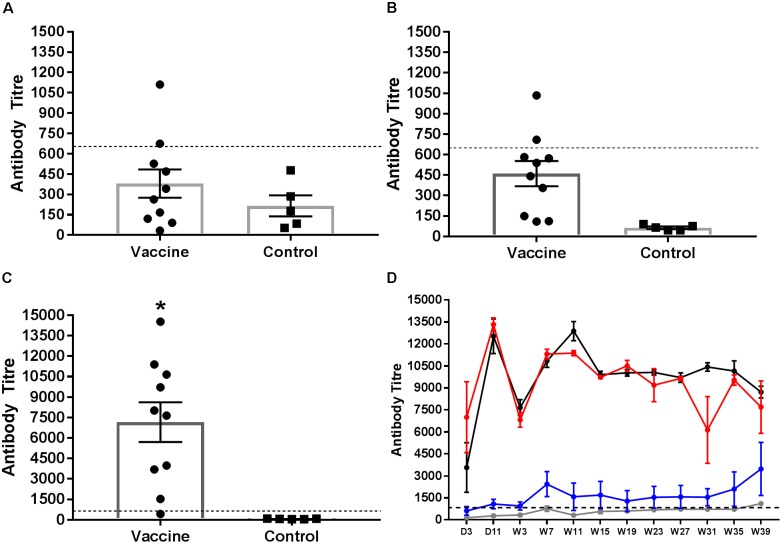
*Salmonella* group B antibody response following vaccination Serum samples were collected and tested for group B LPS antibodies. Samples were taken from birds 1 week after vaccination with STM1. The first STM1 dose was administered by ocular inoculation at day old **(A)**. The second dose was administration in drinking water at 6 weeks of age **(B)**. The final dose was administered by IM injection into the pectoralis muscle at 10 weeks of age **(C)**. Data are presented as mean ± standard error of the mean. The mean antibody titre following the first **(A)** and second **(B)** doses of STM1 vaccine was not significantly different from control unvaccinated birds and was below the positive threshold. Following IM inoculation, the mean antibody titre was significantly higher than control (*P* < 0.001). The post-challenge IgG response from day 3 p.i. to week 39 p.i is shown in **(D)**. Antibody titres from control (uninfected and non-vaccinated) (gray line) and vaccinated only (blue line) birds were not significantly different from each other. Peak antibody titres were observed in the challenged only (black line) and vaccinated and challenged (red line) treatment groups at day 11 p.i. Both challenged only and vaccinated and challenged birds exhibited significantly higher antibody responses than either control or vaccinated only birds (*P* < 0.001). The black hashed line indicates the threshold for positive samples.

Serum antibodies to group B LPS were quantified from chicks 2 weeks after the first and second vaccinations and the final administration of STM1 vaccine in the pectoralis muscle (**Figure 4A**). Data are presented as mean antibody titre ± the standard error of the mean. Antibody titres from vaccinated birds after the first (ocular dose; day old) dose were not significantly greater than control birds (*P* > 0.22, df = 13) (**Figure 4A**). One week after the second (drinking water dose, 6 weeks of age) (**Figure 4B**) dose group B antibodies were significantly greater than unvaccinated birds (*P* < 0.001, df = 13). Antibody titres observed following both STM1 doses, however, were not above the positive threshold for the ELISA. Following the third inoculation into the pectoralis muscle at 10 weeks of age, serum antibody titres in vaccinated chicks increased substantially (**Figure 4C**). The mean antibody titre in vaccinated chicks was 7161.0 ± 8.15 and was significantly higher (*P* < 0.001, df = 13) than the mean titre observed in control birds, 64.15 ± 8.75 (**Figure 4C**).

The *Salmonella* group B antibody response post-challenge with *S.* Typhimurium DT9 was also monitored over the course of the infection trial (**Figure 4D**). The peak antibody response for challenged only and vaccinated and challenged birds occurred at day 11 p.i. A drop was observed in both groups at week 7 p.i. but antibody titres stabilized over the remaining duration of the trial. No significant difference (*P* > 0.50) was detected between these two treatment groups. Birds in the vaccinated only group exhibited antibody titres that remained significantly lower (*P* < 0.0001) than both challenge groups. Over the course of the experiment, titres obtained from vaccinated only birds were not significantly different (P < 0.05) from control birds.

**FIGURE 5:**
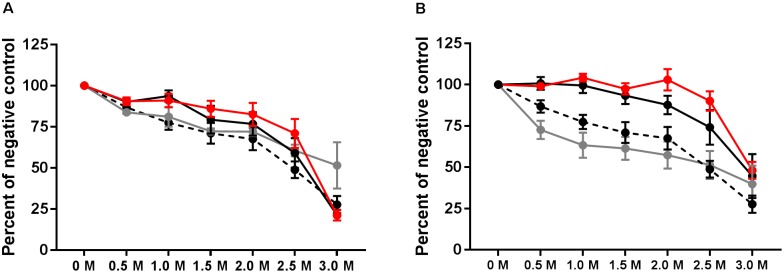
Avidity of group B antibodies. Antibody avidity was tested by washing ELISA plates with varying concentrations of sodium thiocyanate following incubation with test plasma. Avidity was tested at 3 weeks p.i. **(A)** and at week 39 **(B)**. Avidity of final vaccination (at 10 weeks of age prior to challenge) is shown in both A and B by the hashed line. Vaccinated birds (gray), vaccinated and challenged (red), and challenged only (black).

IgG avidity was tested 1 week after the final vaccination, at 3 weeks p.i., and at the end of the experiment at 39 weeks p.i. Relative antibody avidity is represented as percent of negative control. At week 3 p.i., vaccinated, vaccinated and challenged as well as challenged only birds exhibited antibody avidity that did not differ from values obtained from the final vaccination samples (**Figure 5A**). At the 3M concentration of sodium thiocyanate, however, antibody produced after the final vaccination exhibited a higher avidity than the 3 week treatments (**Figure 5A**). At 39 weeks p.i., plasma collected from vaccinated and challenged and challenged only birds exhibited *Salmonella* group B antibody avidity that was significantly greater than the vaccinated only birds for all concentrations except 3.0M (*P* < 0.001) (**Figure 5B**).

### Detection of IgA in Intestinal Mucous

**FIGURE 6:**
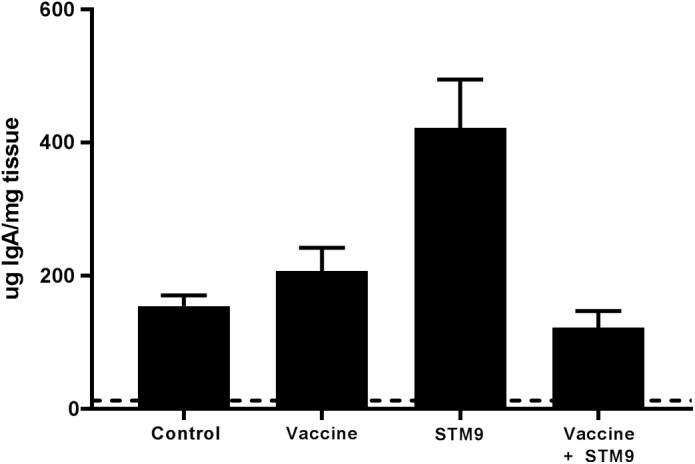
Comparative titres of mucosal IgA. Ileal scrapings were collected at the end of the experiment. Secreted IgA was quantified using a chicken specific ELISA. Mean sIgA titres ranged between 122.3 ± 24.9 μg IgA/mg tissue and 422.8 ± 72.6 μg IgA/mg tissue. The mean sIgA titre observed in the challenged only group (*S.* Typhimurium DT9; indicated as STM9) was significantly higher than all other treatment groups (*P* < 0.05).

Secreted mucosal IgA (sIgA) was quantified from ileal scrapings collected from all birds at 41 weeks p.i. Secretory IgA was detected in all treatment groups (**Figure 6**). Birds challenged with *S.* Typhimurium DT9 exhibited a significantly higher mean concentration of sIgA (422.8 ± 72.6 μg IgA/mg tissue) than all other treatment groups (*P* < 0.05, df = 18, *R*^2^ = 0.6494). No significant difference in mean sIgA concentration was detected between the control (154.7 ± 16.1 μg IgA/mg tissue), vaccinated only (207.4 ± 34.9 μg IgA/mg tissue), or vaccinated and challenged treatment groups (122.3 ± 24.9 μg IgA/mg tissue).

### Detection of *S.* Typhimurium DT9 and STM1 Vaccine by Culture and PCR in Tissues

At week 40 p.i., birds were humanely euthanized and sections of the spleen, liver, ileum, caeca, infundibulum, isthmus, magnum, shell gland, and vagina were collected and processed for bacteriology. A very low rate of *S.* Typhimurium DT9 colonization was observed (**Table 3**). For the ileum samples, only one out of 12 from challenged only and one out of 14 from the vaccinated and challenged groups were culture positive for *S.* Typhimurium DT9. One of 14 caecal samples from the vaccinated and challenge group was *S.* Typhimurium DT9 positive.

**Table 3:** *Salmonella* Typhimurium DT9 detected in tissue samples collected from hens at week 40 p.i.

	Challenged only			Vaccinated and challenged		
Organ	Culture	Enriched culture	PCR	Culture	Enriched culture	PCR
Spleen	0/12	0/12	0/12	0/14	0/14	0/14
Liver	0/12	0/12	0/12	0/14	0/14	0/14
Ileum	1/12	0/12	0/12	1/14	2/14	0/14
Caeca	0/12	2/12	0/12	1/14	3/14	0/14
Infundibulum	0/12	0/12	0/12	0/14	0/14	0/14
Isthmus	0/12	0/12	0/12	0/14	0/14	0/14
Magnum	0/12	0/12	0/12	0/14	0/14	0/14
Shell Gland	0/12	0/12	0/12	0/14	0/14	0/14
Vagina	0/12	0/12	0/12	0/14	0/14	0/14

Tissue homogenates were subsequently enriched for *Salmonella* by culturing overnight in buffered peptone water. Enrichment did not substantially increase the number of *S.* Typhimurium DT9 positive samples (**Table 3**). Post enrichment the number of positive ileal and caecal samples from the vaccinated and challenged group increased from 7.14% to 14.3% and 21.4% respectively. All other samples remained negative.

The STM1 vaccine strain was detected only in one out of 14 (7.14%) caecal samples collected from birds in the vaccinated and challenged group. Post-enrichment increased the number of positive caecal samples to two out of 14 (14.3%). STM1 was not detected in any other tissue samples.

PCR was also conducted on DNA purified from homogenate samples. All tissue samples from both treatment groups were negative for *S.* Typhimurium DT9 (**Table 3**). One caecal sample from the vaccinated and challenged group was PCR positive for STM1; this sample was also culture positive (data not shown).

## Discussion

Previous study of *S.* Typhimurium in layer hens has studied the effects of infection over a limited time frame (Wales and Davies, 2011) or to mid-lay (Pande et al., 2016a). The life span of a commercial layer hen, however, ranges between 60 and 80 weeks. The experiments described here are unique as they were designed to test the efficacy of a commercially available live, attenuated *S.* Typhimurium vaccine using a modified vaccine schedule recommended by the vaccine manufacturer over a 60 weeks period. The primary aim of these experiments was to determine whether the STM1 vaccine could reduce fecal shedding of *S.* Typhimurium and thus reduce egg contamination over a commercially relevant timescale. An additional outcome of this experimental design is the extended characterization of *S.* Typhimurium infection in laying hens.

The vaccination schedule used in this study consisted of three separate doses. The first dose was administered intraocular at day old to mimic the course spray method used at the commercial hatchery. The second dose was given in the drinking water when pullets were 6 weeks of age. The final vaccination administered intramuscularly (IM) in the pectoralis muscle at 10 weeks of age. The IM inoculation has been shown to elicit an enhanced immune response and extend protection (Jackson, 2012; Groves et al., 2016). Additionally, the vaccination schedule used in the current study is based on current industry practice and has recently received Australian Pesticides and Veterinary Medicine Authority approval (Bioproperties, personal communication).

Following the first two vaccinations, serum antibody responses to the vaccine were not significantly higher than unvaccinated birds and did not exceed the assay threshold. Two weeks post-IM inoculation, however, a strong antibody response was observed that was significantly higher than control birds. These results are consistent with previous reports demonstrating that the mode of administration of live attenuated and inactivated vaccines has been shown to affect the strength of the humoral immune response to vaccination (Abs El-Osta et al., 2015; Groves et al., 2016). The avidity assay demonstrated that antibodies produced in vaccinated animals exhibited a similar avidity to *Salmonella* group B antigens as compared to birds challenged with the wild-type *S.* Typhimurium DT9 strain early post infection. While the vaccinated only birds did not differ significantly in avidity between early and late infection time points, at week 39 the vaccinated and challenged and challenged only birds exhibited significantly higher avidity, which may be correlated with the chronicity of infection and the continuous exposure of the bird immune system to *S.* Typhimurium DT9.

Secreted, intestinal mucosal IgA provides a first line defense against oral exposure to bacterial, viral, or parasitic pathogens (Fagarasan, 2006). Forty-two weeks post-infection, birds were euthanized and ileal mucosal scrapings were collected and processed for measuring secreted IgA to determine if vaccination with STM1 had a long term effect on secretory IgA titres. It has been previously shown that secreted mucosal IgA was substantially (albeit not significant) higher in vaccinated challenged birds compared to challenged only individuals (Muir et al., 1998). In contrast, results from the present study found that *S.* Typhimurium DT9 challenged birds only exhibited significantly higher secretory IgA titres than all other treatment groups. Serum IgA has been shown to correlate with levels of secreted IgA in the mucosa (Rose et al., 1981; Brito et al., 1993). It has been previously shown that serum IgA concentrations steadily increase early during infection with *S.* Typhimurium (Beal et al., 2004; Withanage et al., 2005) and stabilize over a longer period and did not significantly change upon re-challenge (Beal et al., 2004). It is challenging to compare previous results with the present study, as there are substantial differences in experimental design. During this study, low secretory IgA levels in the vaccinated group could be attributed to the lack of inflammatory reaction produced by the ST1 vaccine strain. Presentation of an antigen from the intestinal lumen into lymphoid aggregates from the payer’s patches is a prerequisite in the stimulation of IgA response. Antigen present in the intestine of mammals is typically taken into the gut associated lymphoid tissues (GALT) across the specialized epithelium. Such specialized epithelium is not so frequently found in chickens hence, there may be variation in the cell type and mechanisms involved in the induction of IgA response in chicken gut (Muir et al., 2000). Further research is required to investigate uptake of ST1 vaccine from the lumen in avian GALT. Given the importance of the local gut immune response, focus on developing vaccines that are effective at producing a strong mucosal response (Gayet et al., 2017) may be the key to reducing *Salmonella* colonization of the intestine and subsequent shedding in feces. In this study, hens were euthanized at the end of the trial and further studies are required to study secretory IgA response in the gut of vaccinated and vaccinated challenged birds, euthanized at periodic intervals after infection.

Birds were challenged with *S.* Typhimurium DT9 at 18 weeks of age. Unvaccinated birds exhibited blood tinged mucoid feces for several days post-challenge. Clinical signs of disease are not typically associated with *S.* Typhimurium infection in adult layer hens (Pande et al., 2016a). The birds in this study, however, were naïve to *Salmonella* infection and a high challenge dose (1 × 10^9^ CFU) was administered. Pande et al. (2016a) noted similar clinical signs of disease in birds challenged at 14 weeks of age with a different isolate of *S.* Typhimurium DT9. The challenge dose used in the present study was selected, in part, because high bacterial titres are frequently detected in environmental samples (Chousalkar et al., 2016; Gole et al., 2017). By contrast, vaccinated birds, in this study, exhibited limited evidence of disease. Vaccination against *S.* Typhimurium has been shown to provide immunological protection against the systemic spread of the bacteria to the spleen and liver (Hassan and Curtiss, 1994), thus limiting the severity of clinically observable disease symptoms.

The patterns of fecal shedding were monitored for 41 weeks post-challenge with *S.* Typhimurium DT9. Fecal shedding was variable in both challenged and vaccinated and challenged treatment groups over the course of the study. Vaccination had no effect at reducing the number of *S.* Typhimurium DT9 positive fecal samples. Overall, no significant difference was detected between challenged and vaccinated and challenged treatment groups. This result is in contrast to previous reports demonstrating that birds vaccinated with either STM1 vaccine (Alderton et al., 1991) or a *S.* Typhimurium rough strain did not shed bacteria for 35 days post infection (Barrow et al., 1990). Barrow et al. (1990) also reported that *aroA* mutant S. Typhimurium F98 initially reduced fecal shedding of the challenge strain but this effect did not persist. Tan et al. (1997) reported that vaccination *aroA* mutant of *S.* Typhimurium 3860C also did not affect the prevalence of wild-type *Salmonella* in cloacal swabs. These results were irrespective of route of inoculation (Tan et al., 1997). A more recent report showed that vaccination with STM1 did not have an effect on the number of positive fecal samples over a 56 weeks period (Groves et al., 2016).

The effect of vaccination on the abundance of the challenge strain in fecal samples over the course of the experiment was determined using qPCR. No significant difference in the total load of *S.* Typhimurium DT9 was observed between vaccinated and challenged and challenged only birds. Despite the high degree of primer specificity, it should be noted that high resolution melting of amplicons was not performed, thus the detection of non-specific amplification, including primer dimer cannot be precluded. Previous STM1 vaccine efficacy studies have monitored only bacterial colonization and host immune response to vaccination and did not characterize wild-type *Salmonella* load in response to vaccination (Alderton et al., 1991; Groves et al., 2016). Other efficacy trials investigating other *aroA* mutant *S.* Typhimurium strains have also focussed only on change in prevalence and not abundance of the bacteria in fecal samples (Barrow et al., 1990; Tan et al., 1997). Ileal and cecal sections of birds vaccinated with *aroA* mutant of *S.* Typhimurium 3860C remained culture negative for the challenge strain over a 12 month period but evidence of fecal persistence of wild-type *Salmonella* was not investigated (Hassan and Curtiss, 1997). In this study we used both standard PCR for the detection and qPCR for measuring load of wild type *S.* Typhimurium DT9 from feces. The standard PCR was more sensitive than cyber green based QPCR. Hence the standard PCR relatively ruled out false negative QPCR results.

Eggshell contamination with *S.* Typhimurium DT9 was also found to be variable over the course of the experiment. No significant difference in the proportion of *S.* Typhimurium DT9 contaminated eggs was observed between challenged only and vaccinated and challenged treatment groups. Previous studies investigating the effectiveness of the STM1 vaccine have not investigated the effect of vaccination on egg shell contamination. Arnold et al. (2014) evaluated the effectiveness of four vaccination strategies and reported a reduction in egg contamination with *S.* Typhimurium in vaccinated birds. Vaccination with a Δ cya, Δ crp mutant *S.* Typhimurium strain has been reported to completely prevent contamination of both egg shells and egg internal contents with *Salmonella* Typhimurium (Hassan and Curtiss, 1997). On production farms, however, unless a significant reduction in the total environmental load of *Salmonella* is achieved eggs will likely still acquire *Salmonella* spp. on the shell surface in a highly contaminated environment. Gole et al. (2014) reported that positive fecal, egg belt, and dust samples were all highly associated with *Salmonella* contamination of eggs. Furthermore, they demonstrated that the odds of an eggshell becoming positive for *Salmonella* were highest when a feces from a cage was also positive (Gole et al., 2014). It should be noted that, in the present study, no egg internal contents were found to be positive for *S.* Typhimurium DT9. This result is consistent with a recent *S.* Typhimurium infection trial in layer hens (Pande et al., 2016a).

The STM1 vaccine was detected by culture following the second administration of the vaccine in water and during the first week post-challenge with *S.* Typhimurium DT9 in the vaccinated and challenged treatment group. After week 1 post challenge, STM1 was not cultured from fecal samples. Post-challenge, STM1 was intermittently detected by only by PCR in a limited number of fecal samples. Other STM1 vaccine efficacy trials have not monitored the persistence of the vaccine strain only its effect on the host immune response and limiting wild-type *Salmonella* shedding (Alderton et al., 1991; Groves et al., 2011, 2015). Following vaccination, another *aroA* mutant vaccine, *S.* Typhymurium 3860C, was detected by culture for 28 days (Tan et al., 1997). In chicks, an *aroA S.* Enteritidis deletion mutant colonized the liver and caeca but the total load of the mutant strain was significantly reduced between seven and 14 days post-infection (Methner et al., 2011). These results may indicate that the vaccine does not establish a persistent infection within the host. Long term persistence of a live *Salmonella* vaccine in the host is important for horizontal transfer of the vaccine strain within a flock. This is critical in commercial poultry industry because cost remains the major driving factor for adoption and use of new vaccines. In the current study, the data on shedding of STM1 in feces suggests that this vaccine has limited ability for horizontal transfer.

At 60 weeks of age, birds were humanely euthanized and organs were collected for bacteriology to study *S.* Typhimurium DT9 persistence in vaccinated and unvaccinated birds. Spleen and liver samples from all birds were negative for *S.* Typhimurium DT9. This result is in contrast to a recent layer hen trial which demonstrated that spleen and liver samples remained positive for *S.* Typhimurium for 16 weeks post-infection (Sharma et al., 2017). Duplicate ileum, caecal, infundibulum, isthmus, magnum, shell gland and vagina tissue sections were collected and treated with gentamicin to kill any bacteria adhered to the surface. This method ensured that any bacteria cultured would most probably be intracellular. All of these samples were negative suggesting that positive samples likely had *S.* Typhimurium DT9 in the intestinal contents. It has long been thought that *Salmonella* invades and establishes a persistent infection within intestinal cells. It maybe, however, that the bacteria establishes itself as part of the gut microbiome. Modulation of diet and or the use of prebiotics and probiotics could potentially be used to modulate the gut microbiome and thus contribute to limiting *Salmonella* colonization of poultry (Pan and Yu, 2014). Further research in this area is, however, necessary.

## Author Contributions

AM designed the experiments, conducted the animal trial, sample collection, processing, conducted DNA extraction, PCR, ELISA’s, data analysis, and wrote the manuscript. KC designed the experiments, obtained funding, participated in conducting the animal trial, sample collection, processing, and provided input to the manuscript.

## Conflict of Interest Statement

The authors declare that the research was conducted in the absence of any commercial or financial relationships that could be construed as a potential conflict of interest.
